# Engineering Approaches for Programming Agent-Based IoT Objects Using the Resource Management Architecture

**DOI:** 10.3390/s21238110

**Published:** 2021-12-04

**Authors:** Fabian Cesar Brandão, Maria Alice Trinta Lima, Carlos Eduardo Pantoja, Jean Zahn, José Viterbo

**Affiliations:** 1 Federal Center for Technological Education (CEFET-RJ), Rio de Janeiro 20271-110, Brazil; fabiancpbm@gmail.com (F.C.B.); maria.trinta@aluno.cefet-rj.br (M.A.T.L.); 2 Institute of Computing, Fluminense Federal University (UFF), Niterói 24220-900, Brazil; jeanozahn@gmail.com (J.Z.); viterbo@ic.uff.br (J.V.)

**Keywords:** embedded multi-agent systems, IoT, edge computing

## Abstract

The Internet of Things (IoT) allows the sharing of information among devices in a network. Hardware evolutions have enabled the employment of cognitive agents on top of such devices, which could help to adopt pro-active and autonomous IoT systems. Agents are autonomous entities from Artificial Intelligence capable of sensing (perceiving) the environment where they are situated. Then, with these captured perceptions, they can reason and act pro-actively. However, some agent approaches are created for a specific domain or application when dealing with embedded systems and hardware interfacing. In addition, the agent architecture can compromise the system’s performance because of the number of perceptions that agents can access. This paper presents three engineering approaches for creating IoT Objects using Embedded Multi-agent systems (MAS)—as cognitive systems at the edge of an IoT network—connecting, acting, and sharing information with a re-engineered IoT architecture based on the Sensor as a Service model. These engineering approaches use Belief-Desire-Intention (BDI) agents and the JaCaMo framework. In addition, it is expected to diversify the designers’ choice in applying embedded MAS in IoT systems. We also present a case study to validate the whole re-engineered architecture and the approaches. Moreover, some performance tests and comparisons are also presented. The study case shows that each approach is more or less suitable depending on the domain tackled. The performance tests show that the re-engineered IoT architecture is scalable and that there are some trade-offs in adopting one or another approach. The contributions of this paper are an architecture for sharing resources in an IoT network, the use of embedded MAS on top IoT Objects, and three engineering approaches considering agent and artifacts dimensions.

## 1. Introduction

The Internet of Things (IoT) is a network that links objects on the Internet, enabling large-scale data sharing and remote control of physical devices to implement distributed systems [1]. Once connected to the IoT, physical devices become a network object, and their resources could be available for other interested persons or even objects. One of the IoT’s challenges is how to interact with different heterogeneous devices using different technologies and architectures to communicate with the network and expose their resources.

Moreover, cognitive embedded systems on top of devices at the edge of a system could add autonomy, intelligence, and improve data processing and decision making. A Multi-Agent System (MAS) is a system composed of cognitive agents capable of acting pro-actively and autonomously and communicating with other agents and entities to achieve common or conflicting goals in a situated environment, which could be simulated or a physical one [2]. Agents can perceive the environment to collect perceptions from it using sensing mechanisms such as sensors and act upon it using actuators in the case of physical environments. MAS are often used as cognitive embedded systems at the edge of some systems and architectures [3,4,5].

Usually, the works integrating IoT solutions in a physical environment using embedded agents or MAS in the edge generally adopt centralized architectures [6], do not offer heterogeneity in hardware or software, or are tied to a specific domain [7]. Some proposed architectures integrating IoT and MAS could overcome some of these issues. The Resource Management Architecture (RMA) [8] provides a methodology for exposing IoT Objects enhanced with embedded MAS in the IoT, guaranteeing the hardware heterogeneity and abstracting technical details of the device from applications.

However, when agents try to access information available in physical environments by interfacing to sensors and actuators, they could be overloaded in perceiving and processing a massive amount of perceptions available. Every piece of information captured by agents from the environment using their sensors is considered a perception. Agents could use these real-world perceptions coming from sensors for activating plans or keeping the mental state of some situation. Therefore, the deliberation process will possibly be affected by techniques and strategies to deal with these issues, such as applying perception filters [9], dealing with the active perception process [10], or adopting sensors and actuators as artifacts in a MAS [4]. Since artifacts are physical or virtual entities that provide functions or services that agents can use to achieve their goals [11], we assert that artifacts could be adapted, improved, and combined to provide engineering options when creating MAS on top of IoT Devices at the edge.

Thus, the objective of this work is to propose three engineering approaches for programming agent-based IoT Objects at the edge of a system using an extended and improved version of the RMA. The engineering approaches use an embedded MAS to control an IoT Object, allowing specialized tasks for agents. Some of them can be responsible for communication, other for interfacing hardware, or any task necessary to achieve their collective goals as an IoT Object. It brings advantages and performance gain in the deliberation process compared to traditional approaches where only one agent controls all IoT Objects’ functionalities. In addition, an embedded MAS can employ virtual artifacts to access hardware or communicate with RMA since it can create its own internal and virtual environment, representing a real physical environment inside the IoT Object. The embedded MAS is independent, so its agents can communicate with agents from other embedded MAS. It can also share information with the RMA, which will be available to be consumed by clients in an application layer of the adopted architecture.

Each approach varies in how the embedded MAS in IoT Objects behave while accessing a cyber-physical environment. The first approach considers agents interfacing hardware directly and processing information to send improved knowledge to the IoT architecture. Artifacts play a major role in the others engineering approaches. A virtual representation of sensors and actuators is assembled in IoT Objects as Artifacts, named in this paper as Physical Artifacts since they directly access physical resources. They can share information with the embedded MAS or directly to the RMA to relieve some agents’ processing costs.

In this paper, the Agents and Artifacts are programmed using JaCaMo [12], which combines agent and environment dimensions in the same framework. It uses the Belief-Desire-Intention (BDI) [13] model as a cognitive model. BDI agents can achieve goals based on desires and intentions, activated by beliefs acquired from the environment (perceptions) or communication with other agents (messages). Desires and Intentions are achieved through plans and actions. The RMA employs the ContextNet [14] in all its layers for establishing an IoT Server, a shareable data infrastructure, and a scalable network. The contributions of this paper are the refactored and extended RMA for sharing IoT Objects’ resources for clients in an IoT network, the capability of adopting embedded MAS, on top of IoT Objects, as the cognitive system to provide intelligence, pro-activity and autonomy, and give designers options in how to engineer IoT Objects by adopting three strategies to program the embedded MAS in the RMA. Communicator agents and Physical and IoT Artifacts are presented for the JaCaMo framework and an extended version of RMA.

This paper is structured as follows: Section 2 shows some related works; Section 3 presents the RMA; in Section 3.1 some definitions necessary to understand the paper are introduced; in Section 3.2, the engineering approaches using the extended RMA are proposed; Section 3.3 presents the RMA extension and the approaches’ implementation; in Section 4, a case study is presented adopting a home garden scenario; and Section 5 presents the final considerations.

## 2. Related Works and Motivations of This Work

The IoT has motivated several works in distributed computing intending to share the resource information of connected objects aligned with the advance of hardware technologies. Mainly, agents have been deployed on microprocessors, such as Raspberry Pi [15,16]. These agents may be either static or capable of moving from board to board, creating a network and a distributed MAS. Concerning this point, static agents could be overloaded if the amount of information coming from sensors exceeds its processing power and mobile agents will also rely on storage and memory capacity. Even though agents are autonomous and proactive, they cannot perform two actions at the same time in two different actuators or sensors, for example. An Embedded MAS could employ specialized and dedicated agents for splitting the responsibility of achieving a goal: for example, agents to deal with communication or a small group of sensors.

Many challenges arise when employing embedded agents or even embedded MAS. Agents have to deal with the heterogeneity of hardware (sensors, actuators, boards, etc.) and sources (other agents or MAS, applications, etc.). There is also a direction to create decentralized MAS to insert intelligence in IoT systems [17]. One of the main challenges is how to deal with the heterogeneity of IoT devices and performance. It is not a simple task to provide an architecture to integrate and control smart devices in IoT [18]. It becomes worse when considering agents and MAS since they could be logically developed using different frameworks or adopting different communication protocols. Moreover, an embedded MAS can be considered a fully autonomous embodied agent when interfacing hardware, such as a robot that employs an MAS. Even in robotics, cooperation between robots is a challenge to be faced since many of them are pre-configured in design-time [19].

In addition, some approaches search for autonomy and some intelligence level in the edge of the network by adopting architectures [20]. There is an architecture that aims to provide intelligent and adaptable home systems using the Fog computational concept [21], which does not focus on the agent approach. It address some issues, such as the limitation of resources in IoT devices, data volume, heterogeneity, loss of connection, and others. It has two layers: the Cloud Layer and the Fog Layer, where the former is responsible for the flow, processing, and analysis of data. The latter implements some of these features to distribute the responsibilities and remove the overload that could exist in the Cloud Layer. However, the architecture is dedicated to data processing and does not provide a heterogeneous way to address the gap between hardware and agents.

When considering agent-based IoT architectures, some solutions provide services, communications, and configurations for dedicated applications and domains such as shopping malls [20], cattle [22], co-working buildings [23], and a manufacturing scenario in an industrial environment [24]. In addition, agent-oriented programming languages such as Jade [25] and Jason [10] play a major role in several solutions [26,27,28]. The Agent-based Cooperating So (ACOSO) works as an IoT middleware to provide agents as devices and several agents as an MAS [29,30]. The ACOSO supports the development of cognitive MAS for IoT. Basically, each smart object is abstracted to a cooperating Jade agent. It runs over a three-layered architecture: application, transport, and net and physical, where agents can manage sensors and actuators; reason using local and distributed databases; and access a communication system to interact with smart objects and other structures. These architectures centralize the MAS on a server side or adopt one agent per sensor. There are some issues with centralizing the cognitive system in a server, such as technological dependency. Some solutions would not work correctly if the agent lost communicability with other agents. Embedded MAS should provide real autonomy, pro-activity, and independence without considering centralized technologies. They should send information to an IoT server but still monitor and reason at the edge of the system by manipulating their sensors and actuators.

Particularly, a multi-agent architecture proposed for the fast and efficient management of data coming from basic hardware devices in the IoT provides services in a layer to fulfill requirements of a given environment [28]. There is local processing not to transmit all data produced in the edge to the cloud server considering reductions in time and the cost of processing. Embedded reactive agents manage the information generated from the IoT Systems, and they communicate to each other adopting the MQTT protocol. The embedded multi-agent that manages the industrial wireless sensor network has embedded agents in its nodes [27]. It considers active nodes where the data will only be transmitted if requested. Otherwise, passive nodes use an acknowledged time-based data transmission process. Table 1 shows a brief comparison of all related works. It considers the platform used in each solution, the domain applied or if it is generic (it could be reused and it is not tied to a specific domain), and the agent composition where it was employed one agent per device or an embedded MAS.

In this paper, we propose an uncoupled and decentralized architecture to employ MAS on top of IoT Objects and mechanisms to allow communication between hardware, agents, and the IoT network, abstracting all the technical details from users and designers and also between the the proposed architecture’s layers. The Embedded MAS have dedicated agents to deal with sensors and actuators, others to communicate with the IoT server and devices, and finally, some agents for general purposes. As said before, employing multiples and specialized agents at the edge of the system could reduce bottlenecks in accessing sensors and actuators and even reduce processing time on the server side since it tends to be less requested. The architecture is generic enough and prepared to couple with any domain since they follow a simple protocol for exchanging messages between edge objects and the IoT server.

Although the novelty of using embedded MAS as a cognitive system to provide autonomy and some intelligence for devices that can operate independently from centralized servers and still reason and communicate, it brings challenges that different programming strategies could tackle. Then, we present three novel engineering approaches to build IoT Objects embedded with MAS, including the agent dimension, and the endogenous environment dimension (artifacts), using the BDI model. An engineering approach could perform better than another depending on the solution domain that the designer must create. For example, it is possible to merge active nodes (from the agents’ point of view) and passive nodes (from the IoT server’s point of view) into a device. This configuration could be interesting in a situation where agents should reason and give fast answers in the edge without communicating to the server-side (active node) and the device still needs to transmit data to the server (passive node).

## 3. Resource Management Architecture

The RMA is an architecture for sharing the devices’ sensors and actuators on the IoT using a model that allows exposing these resources to be consumed by clients [8]. For this, the architecture maintains information about these sensors and actuators in a centralized layer. The RMA was designed inspired by the Sensor-as-a-Service (SaaS), an emerging cloud computing model allowing sensors and actuators to share data on the Internet of Things to be consumed and commercialized by applications and third parties [31].

The architecture allows any person to adopt devices in some environment to access information and autonomously or remotely control it. The device works pervasively by means of their sensors and actuators managed by an embedded system, which provides all the functionalities for interfacing hardware, receiving commands, and sending data to an IoT server. The owner of a device places it in a room, for example, and then configures it to connect to a server-side application to share sensory information and to be virtually available for clients applications and other users, or just privately. Sometimes the owner might want to keep all his devices available for himself, maybe because it contains personal or sensible data (i.e., a hospital room). Otherwise, it could be publicly available for anyone who wants specific information, such as the temperature or the weather condition in a particular spot.

All devices sends their identification, composition, localization, and available functionalities when connected to the IoT Server. The server-side application stores all these data in a database and maintains them while the device is connected. If the device goes offline for any reason, the server-side application turns the device unreachable until it reconnects. In addition to managing the connection and reconnection, the server-side application is also responsible for managing all the data received from devices’ sensors and clients’ command requests (i.e., turn the lights of a room on or off). The same database stores the devices’ data to be consumed whenever a client desires, and it redirects the command requests to the proper device, which holds the target actuator.

**Figure 1:**
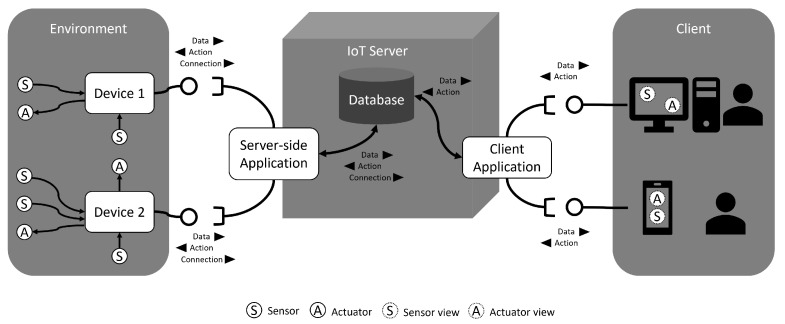
The RMA overview.

Clients access applications to consume information from virtualized devices. They can remotely control them by accessing mobile phones, web services, or applications, which connect directly to the database where the data were previously stored. These client applications also interact with the server-side application to send command requests to be performed in devices. For example, if someone forgets to turn off a light when leaving his house in the morning, it could verify the light status and send a command request to turn off the light at anytime. The Figure 1 shows the RMA overview.

The RMA plays an essential role in defining the engineering approaches since it provides a decoupled layered architecture that could be explored to implement edge computing using MAS. The RMA allows multiple device connections to share data, communicate with each other, and all data shared using the RMA can be consumed by application clients, reinforcing the potential of using it. By adopting MAS at the edge of the system on top of devices, some data processing can be performed directly on the device, decreasing the server-side usage for these situations. It could also reduce the response time for any situation captured by sensors since agents can act proactively.

The use of MAS on top of IoT Systems offers several challenges that few works tackled during the years. The MAS should be autonomous and interface controllers, sensors, and actuators. It also needs to communicate to a server-side application and other devices. In addition, it must observe and provide constructions for all the dimensions of the agent paradigm, which facilitates integration between the environments and the MAS architecture.

The proposed engineering approaches provide ways for how to embed an MAS on top of devices considering hardware interfacing, communication, performance, and the agent’s dimension. These approaches consider specialized agents created and adapted to deal with sensors and actuators interfacing and IoT communication. In addition, artifacts could play the same role as these agents, increasing the possibilities of designing the embedded MAS. Depending on the problem addressed, one could employ agents, artifacts, or both, which is a novelty when considering embedded agents and IoT systems.

### 3.1. Definitions and Working Details

To fully understand the RMA, it is important to define its central concepts, components, and how they work together. The architecture works around the notion of Devices that communicate with the server-side application to provide Resources such as environmental sensors and actuators. Then, we define Device and Resources as follows:

**Definition** **1.**  *A Device is a composition of hardware parts and an embedded system built together with a particular purpose for interacting upon some physical environment. These hardware parts are electronic components responsible for sensing and acting in the physical environment where the Device can be situated.*

**Definition** **2.**  *Resources are the sensors and actuators used in Devices.*

For example, in a home garden scenario, Devices could exist with proper sensors and actuators to monitor all information about a plant, such as soil moisture and weather prediction. Then, sensors are responsible for collecting data from the physical environment, and actuators act by performing actions that could change the physical environment. Moreover, the home garden owner could activate an irrigator from a distance by sending commands to the Device using a client application. Then:

**Definition** **3.**  *Commands are all actions that can be performed by a Resource when it is an actuator.*

The Device uses an embedded system, which may reason or not, to control, process, and momentarily store and share the Resources’ data using some communication infrastructure. Devices are heterogeneous, which means they can use different technologies for software, controllers, boards, and Resources. A non-cognitive system is responsible only for exchanging data between the hardware and the IoT and consequently it does not perform decision making in the edge of a system. Conversely, a cognitive system can offer reasoning and decision making for the edge of a system, and it could also send improved information instead of just replying to data coming from sensors. When adopting the agent approach, for example, agents can reason and interact with each other based on information gathered from a physical environment.

**Definition** **4.**  *An Embedded MAS is an agent-based system embedded in a device and responsible for the device’s autonomy, pro-activity, and communication by controlling and accessing actuators, sensors, and communication infrastructures.*

In the home garden scenario, an IoT Object embedded with a non-cognitive system will send the raw data of the soil and local temperature to an IoT server. It will receive commands from a distance of the home garden owner to be executed. In cognitive-controlled IoT Objects, agents of an embedded MAS could access these data and reason about the necessity of irrigating the soil if it is dry without waiting for the owner’s command. Then:

**Definition** **5.**  *An IoT Object is a Device connected to exchange messages specifically with the RMA and to share its Resources with application clients.*

The RMA is divided into three layers:

- Device Layer: comprises IoT Objects, which can (i) connect and register in the RML as part of a specific physical environment when it starts running; (ii) share all data of its Resources with the RML, and; (iii) receive Commands to be performed in the physical environment.
- Resource Management Layer (RML): is responsible for registering IoT Objects’ primary information and their Resources’ data, exposing IoT Objects on an IoT network to be consumed by clients, and receiving Commands from clients and redirecting them to the specific IoT Objects. The RML comprises the Resource Management Component (RMC), which handles the IoT Objects’ registering, Resources’ data sharing, and Commands received from the Application layer. In addition, RML is composed of the Virtualized Components Database (VCDB), a database responsible for storing data from all IoT Objects and physical environments. The Environment is the virtual representation of a physical environment inhabited by one or more IoT Object. Furthermore, every Command request that arrives for the RML is forwarded to the specific IoT Object.
- Application Layer: Client applications access the RML to consume the virtualized Resources as a service. The home garden owner can interface RML using an application, for example, to monitor a specific physical environment (a living room) or resource (a specific plant), and send back Command requests to be performed at IoT Objects actuators. These applications can be web services, middleware, and mobile and desktop applications, including MAS applications.

Considering the home garden, when the owner turns on the IoT Object, it connects and registers itself in the RML in a pre-defined virtual environment. For example, the IoT Object could be configured to be situated in a living room or a balcony. Once it is connected and registered, it can send soil and temperature data to the RML, which stores them to be consumed by the home garden owner. In addition, it can receive commands to turn on and turn off the irrigator from the owner. In this case, the RML redirects the commands to the specific IoT Object.

RMA abstracts technical details from IoT Objects and the Application layer in RML. Then, clients can access and consume data from different IoT Objects without knowing which kind of hardware was employed in the design of the IoT Object. The same is valid for the IoT Object’s software side. It is indifferent to the RML or even the Application layer if the embedded system uses a cognitive system or not. The RML gathers data and stores them in the VCDB while redirecting Commands for IoT objects to perform at actuators. This communication is based on a text-based protocol for exchanging messages between layers.

When applying an embedded MAS, it is possible to provide some reasoning on the edge of the IoT Object. Since agents can reason about the data collected from sensors—instead of just replying to them to the IoT cloud instance, the RML—some actions can be performed autonomously at the edge of the system. Moreover, agents can learn or teach other agents from the direct interaction with other IoT Objects since they can share beliefs, desires, and plans. For example, if the owner of the home garden and an IoT Object enhanced if an embedded MAS acquires a new sensor for its IoT Object, it could learn from another IoT Object which shares the same type of sensor by exchanging messages and plans.

However, RMA does not provide all mechanisms and approaches combining the IoT and MAS to create effective systems since it does not consider all possible constructions of the multi-agent approach, such as artifacts at the environment level. Artifacts can improve the modeling by adding an abstraction in representing sensors and actuators that could directly impact the agents’ performance when accessing and processing perceptions coming from these resources. In the next section, we refactor the current RMA to expand design possibilities using embedded MAS and explore them considering agents and artifacts—or even agents’ societal layers—on the edge of IoT systems.

### 3.2. The MAS Engineering Approaches

The RMA is composed by three independent layers that provides all the necessary concepts and structures for creating a network of IoT Objects capable of sharing resources for clients applications. The Device layer is where the design of IoT Objects and the engineering approaches of the system happen. It includes defining the hardware technologies employed, and the embedded systems programming (reactive or cognitive, for example). The strategy adopted by the designer may influence the way in which the system deals with the physical environment while it is sensed and how to interact with the top layer to share its sensing information.

**Figure 2:**
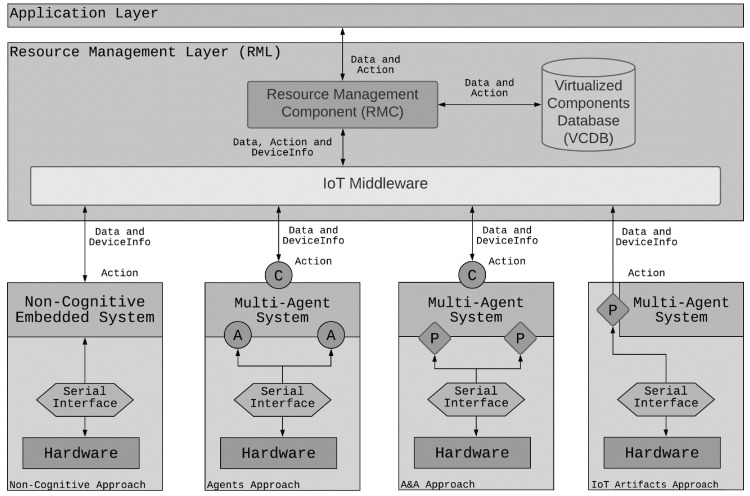
The extended Resource Management Architecture (RMA) and the three engineering approaches in the Device layer: the Agents, Agents and Artifacts (A&A), and the IoT Artifacts Approaches.

In Figure 2, it is possible to observe the Device Layer composition, including the new proposed MAS engineering approaches (Agent, Agent and Artifact, and IoT Artifact). Independent from the engineering approach adopted, each IoT Object is composed of at least one microcontroller (hardware) connected to several sensors and actuators responsible for sensing and acting in a physical environment. The microcontroller is interfaced by a double-layered serial interface, which manages the message exchanges between the hardware and the Embedded System.

The Non-cognitive approach for designing IoT Objects in the RMA uses an embedded system to interface the physical environment and communicate with RML, resulting in an approach that does not provide autonomy and pro-activity during the decision-making process. It works only as a data repeater sending the data gathered from sensors to the RML. Then, we realized that is important to flexibilize the options that the designers could employ to produce solutions in the edge of the system. The three engineering approaches present different ways of including cognition using MAS. In a system composed of several IoT Objects, each one of them can adopt its own approach. Since the layers are uncoupled and use text-based communication, the adopted technology is irrelevant for the functioning of the architecture. We describe the three engineering approaches as follows:

- Agent Approach: it uses an MAS as the embedded system that is capable of processing the gathered information from sensors and decide about the Command request execution coming from the RML instead of transferring this responsibility to upper layers. For this, the MAS comprises Physical Agents capable of interfacing the physical environment and a Communicator Agent to exchange messages with RML and communicate with other IoT Objects. Once the information is stored in the RML, it can be consumed by clients.
- Agent and Artifact (A&A) Approach: it works in the same way as the Agent approach but employing Physical Artifacts instead of Physical Agents for sensing and acting upon the physical environment. These artifacts access the sensors’ values as observable properties and control actuators using operations accessible to any agent. Therefore, a specific type of agent to interface the Hardware is not necessary. The Communicator Agent maintains its role in this approach.
- IoT Artifact Approach: as in the A&A approach, some artifacts continue to collect data from sensors and operate actions in actuators. However, some artifacts themselves communicate directly with the RML and are then named IoT Artifacts. Therefore, there is no need for the Communicator agent anymore. In this approach, the MAS is only responsible for the reasoning in the edge by accessing the observable properties of available artifacts and operating them.

The engineering approaches are transparent to the garden owner in the home garden scenario since the differences are purely technological considering the agents adopted. For example, in the three cases, the Embedded MAS of the IoT Object situated in a plant could verify autonomously if the plant needs water. In addition, the owner can send actions to be executed (e.g., turn on the irrigator) by the IoT Object. In this case, the only difference is if agents or artifacts will treat the action request. Then, one can adopt the approaches considering the available hardware, the system domain, the expected performance, and the IoT Objects behavior in the physical environment. Then, it is important to analyze what each approach can offer and the differences between them.

In the Agent approach, as only one type of agent—the Physical agent—collects all values from the physical environment, it needs to transfer all the gathered information to other agents that use these values to interpret and improve the understanding of what was sensed before sending them to the RML. Moreover, some agents can use these interpretations to reason about the hardware controlling and send their conclusion to Physical agents that act in the physical environment without waiting for the interference of third parts (clients applications by the RML). In addition, actions coming from RML will only be executed if the MAS endorses and forwards them to actuators. The communicator agent allows the embedded MAS to communicate with the RML for registering the IoT Object, sending Data, and receiving Actions. Sometimes, continuously sending data to the RML could be unnecessary or costly. Therefore, it is up to the IoT Objects to decide whether or not to send information to the RML.

However, this approach can generate some performance problems since the entire MAS depends only on the Physical Agents to collect information from sensors and send action commands to actuators. Thus, depending on the problem tackled, it can overload these agents, and some bottlenecks would arise since the data are captured in every execution of the agent’s reasoning cycle, leading to a constant interfacing with the Hardware. It could be expensive considering energy efficiency, which is also a concern in embedded systems.

Adversely, in the A&A approach, any agent can access the artifacts anytime depending on the agents’ needs and the availability of artifacts. It is important to remark that two agents cannot access one artifact at the same time. Although these kind of artifacts could unload agents that interface hardware, some information processing may still be affected because it still has to be collected, processed, and sent to the RML by communicator agents. Depending on the application domain, a hybrid approach could be interesting where agents could even process data and act in the hardware by accessing artifacts and the sensors’ information would be sent directly to the RML by artifacts instead of Communicator agents.

In the IoT Artifact approach, the Communicator Agent is not responsible for communicating with the RML and there are no agents interfacing the physical environment, thus the MAS does not interfere in how data is exchanged between the IoT Object and the RML. However, agents can still collect data from the sensors and perform actions on the actuators using artifacts. In this case, agents access data from the artifact’s observable properties, and actions are executed when agents perform artifact operations. Then, agents can reason and produce internal conclusions to control the IoT Object at the edge of the system. However, raw information will be sent directly to the RML. Therefore, the IoT Artifact approach should be used when an IoT Object just needs to share raw data and perform clients’ actions directly in the RML, and it is still necessary to employ a certain degree of autonomy and pro-activity in the edge.

As said before, it is possible to employ in the same system all three proposed approaches in different IoT Objects, and it is up to the designer when to adopt one or another approach. The designer must observe the latency of the information according to the domain of the application, the response demanded by the application layer, the complexity of the information (raw or reasoned data), and the autonomy of the IoT Object for example. In the following sections, we describe the RMA refactoring and extension, and we detail some behaviors of the architecture.

### 3.3. Extending the RMA and Implementing the Approaches

The RMA provides a way to expose IoT Objects’ resources accessible to client applications hiding technical details of hardware and software. The Device layer comprises all IoT Objects in the edge of a system. Previously [8], the Device layer had no practical way to create an IoT Object, allowing the embedded MAS to interact with the RML. Therefore, RMA needs mechanisms to support the three proposed approaches considering embedded MAS in the edge in terms of generic domain, performance, and data availability.

In the Agent and A&A Approaches, the Communicator Agent is responsible for communicating with the RML from the Device Layer. This agent needs to adopt a communication infrastructure to connect, send information, and receive Commands from the upper layers. Since the Communicator Agent concept [3] was created for allowing two distinct MAS to communicate, it does not consider communication with RMA.

In the IoT Artifacts approach, Physical Artifacts need to send and receive information for both MAS and RML. In the A&A approach, artifacts are only able to interface hardware and agents take place in reasoning and communicating with RML. Then, it is necessary to adapt and implement all those behaviors in Communicator Agents and Physical Artifacts to communicate with RML for creating our proposed approaches.

#### 3.3.1. The Extended RMA

Despite the three approaches being employed specifically in the Device layer, the RML also impacts in their functioning. Then, all possible constructions of the proposed approaches using RMA are defined in a general class model comprising all levels. An IoT Object needs its own independent cycle from other layers that is capable of dealing with a cognitive embedded system or not, as well as IoT Artifacts. In addition, algorithms for initializing and registering IoT Objects are necessary for dealing with the data transfer to RML. The RML also needs a mechanism to maintain historical data in some domains where it is necessary to inform client applications of past sensors’ values. It is expected that RMA will gain more robustness to deal with IoT Objects improving the message exchange process between the Device Layer and the RML, and adopting a NoSQL database based on documents. Thus, we present contributions in (i) the class model, (ii) the IoT Object’s initialization process and cycle, (iii) the database technology, (iv), and the protocol used to exchange text information.

**Figure 3:**
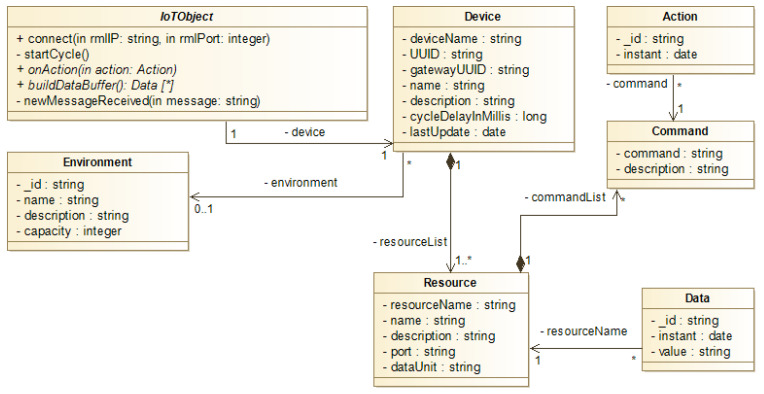
The extended RMA model.

The class model allow defining how Resources, Commands, Devices, and IoT Objects are related in some Environment, as well as the format of messages that are persisted in VCDB as Data and Actions. Figure 3 shows the class diagram of the RMA, its main attributes and methods, and how these classes are associated with each other. Then, a Device is associated with an Environment, and this Environment can have a set of Devices allocated to it; in addition, a Device has one or more Resources. Each Resource can generate Data representing the sensors’ readings and receive Actions representing the actuators’ Commands. In this case, actuators can have one or more Commands that define the possible hardware operations. Both the Data and Action models provide the historical storage of a given resource in the RML.

Finally, an IoT Object is a Device capable of communicating with the RML and both have the same composition—hardware parts and an embedded system—as said before. Technically in the model, a Device class holds all attributes for identifying any device in the RMA. However, as only IoT Objects can connect and communicate with RML, it is necessary to use the IoT Object class. Thus, all IoT Objects are Devices implicitly. Each class is detailed as follows:

- Environment: It represents a physical environment where one or more Device can be situated in. It has a *name*, a *description*, and the *maximum capacity* of IoT objects in the physical environment.
- Device: It represents the hardware composition with one or more Resources and an embedded system. The Device class is identified by a *deviceName*, and has a *name*, which is the device’s nickname accessed by application clients and it does not necessarily needs to be unique; a *description*, which holds basic information about a Device; a Universal Identification (UUID) in the IoT network (*UUID*, a *gatewayUUID*); a delay time between sending data to the RML (*cycleDelayInMillis*); and a date that represents the time of the last update of this Device in the RML (*lastUpdate*).
- Resource: It represents the IoT Objects’ sensors and actuators in the RMA. As in the Device, the *resourceName*, *name*, and *description* identify, nickname, and holds basic information about Resources, respectively. It also has a serial port for communication with the IoT Object’s microcontroller (*port*) and a measurement unit of the information coming from sensors or actuators (*dataUnit*). Every actuator must have at least one Command associated.
- Command: It represents an operation that a Resource can execute when it is an actuator. For example, if a motor is a Resource, it could have Commands “on” and “off”. A Command has as attributes a command name (*command*) and a *description*.
- Data: It represents a value measured in a Resource according to a unit of measure (*dataUnit*) in an instant of time. Data references only one Resource and it represents values of any type, including nominal or quantitative values.
- Action: It represents a Command execution request that must be performed by an IoT Object’s actuator. For this, it is necessary to address the Command, the respective Resource, and the Device where the Action needs to be performed.
- IoTObject: It defines the initialization process of the IoT Object, its cycle, and the Action’s execution. The initialization process connects the Device to the RML, but if this Device does not exists in VCDB yet, it is registered. The cycle is the process that builds a Data buffer and sends it to the RML to be stored by VCDB and consumed by clients. The action’s execution defines how Actions will be performed in the hardware. The *void connect* (*String rmlIP, int rmlPort*) method performs the connection request to the RML, and the confirmation arrives in the *newMessageReceived* (*Message message*) method as a message. If the Device is connected, the cycle starts. The *void startCycle*() method is a loop that gathers Resource’s Data—using *List<Data>buildDataBuffer*() *method*—and sends them to RML. The *void onAction* (*Action action*) is responsible for effectively executing the Action based on the approach chosen by the system’s designer. Finally, each cycle can be delayed using a predefined time (*cycleDelayInMillis* in Device) considering that some Resources do not necessarily vary their values in a short period of time to be constantly updating the RML.

The starting point of RMA is the Device layer since the RML virtualizes IoT Objects and clients look up for specific Resources in some Environment. Then, for the whole architecture to work, IoT Objects need to be registered in the RML before anything else. Once there is an RML instance on the server side, IoT Objects need to connect and register themselves to start sending Data and become available for clients’ Actions. IoT Objects and Devices can operate completely independent of any architecture, since they employ embedded systems with different cognitive levels. When applying embedded MAS, some of server-side processing can be transferred to the edge, increasing the IoT Object’s autonomy.

The IoT Object’s initialization process is represented by the Algorithm 1, which is responsible for connecting it in the RML. Firstly, the IoT Object informs its basic identification information, resources, and commands before sending any resource data to the upcoming layers. In case of the first time that the IoT Object connects with the RML, it will be registered in VCDB; otherwise, the existent IoT Object will be updated. The IoT Object is mounted based on information extracted from the configuration file, which holds all information provided by the IoT Object designer: identification, resources, and commands. In addition, the IoT Object must acquire an available RML’s IP address and port, usually informed by the designer as well.

The IoT Object needs an UUID for uniquely identify it in the RML. This identification is generated by the RML the very first time the IoT Object connects itself. If it is not the first time, the IoT Object retrieves the UUID from previous connections before trying to reconnect. Then, the IoT Object sends the connection request to RML—using all this information—and waits for the acknowledge message. The RML verifies whether is the first IoT Object’s connection or not. In case of the first connection, the RML generates the UUID and stores the IoT Object’s information in VCDB. Otherwise, the RML returns the existing UUID and updates the IoT Object’s information. In both cases, it sends the acknowledge message back. After the end of this process, the IoT Object is able to start its cycle for sending Data to the RML and receive Actions.
**Algorithm 1.** The IoT Object’s initialization process algorithm.  1:**procedure***initialize*(ip, port, configurationFile)  2:    *rml* ← *connection*(*ip*, *port*)  3:    **if** *UUID* == *null* **then**  4:         *UUID* ← *rml.askForUUID*()  5:    **end if**  6:    *device* ← *readDeviceFile*(*con f igurationFile*)  7:    *acknowledge* ← *rml.connect*(*UUID, device*)  8:    **if** *acknowledge* **then**  9:         *startCycle*(*rml*)10:    **end if**11:**end procedure**

Each IoT Object has a cycle (Algorithm 2) that starts when an acknowledge message arrives from the RML. The cycle is responsible for performing Actions coming from upper layers and sharing Resources’ Data with the RML while it is connected. Firstly, The *startActionListener* method activates a listener that detects the arrival of Actions from the RML. For every message arriving, the IoT Object sends the requested command to the microcontroller using the *onAction* method. In each step, a list of Data is retrieved by the *buildDataBuffer* method and then sent to the RML. Finally, the cycle could be delayed by assigning a value in milliseconds using the *cycleDelayInMillis* attribute. Depending on the system’s domain, the designer can use the *cycleDelayInMillis* to regulate the Data latency of an IoT Object while communicating with the RML.
**Algorithm 2.** IoT Object’s Cycle.1:**procedure***startCycle*(rml)2:     *startActionListener*()3:     **while** *connected* **do**4:          *onAction*(*action*)5:          *data*[] ← *buildDataBu f f er*()6:          *rml.send*(*data*[])7:          *wait*(*cycleDelayInMillis*)8:     **end while**9:**end procedure**

The Algorithm 3 describes how the RMC deals with new messages coming from IoT Objects and Clients. The RMC is a specific component that deals with all requisitions and messages that the RML receives. Then, the IoT Object sends its basic information (identification in the network, name, description, and delay in milliseconds), and a list of available Resources and Commands. It also sends messages containing Resources’ Data from their sensors. The Clients send Action requisitions addressing the IoT Object that will perform it. Therefore, each message received is decoded to identify its proper treatment and forwarded to the specific destination (IoT Objects or the VCDB).

In case of the IoT Object’s basic information, Resources, and Commands, the RMC will send an acknowledge message back to the IoT Object. If it is performing its first access, the RMC will register all the IoT Object’s information in VCDB and send back an auto-generated UUID. In case of Resources’ Data, the RMC will register these Data and update the time and date of this latest access in VCDB. Finally, in case of Client’s Action, the RMC stores this message in VCDB, identifies the respective addressee, and sends this Action to the target IoT Object.
**Algorithm 3.** RMC algorithm for receiving messages.  1:**procedure***newMessageReceived*(message)  2:      **switch** *message* **do**  3:           **case** *deviceIn f o*  4:                 **if** *isNotRegistered*(deviceIn f o) **then**  5:                 *registerDevice*(*deviceIn f o*)  6:                 *send*(*UUID*)  7:            **end if**  8:            *sendAcknowledge*(*deviceIn f o*)  9:          **case** *data*[]10:            *saveInVCDB*(*data*[])11:            *setLastIoTObjectAccess*(*date*)12:          **case** *action*13:            *saveInVCDB*(*action*)14:            *device* ← *action.getTarget*()15:            *sendAction*(*device, action*)16:**end procedure**

The algorithms describe how IoT Objects and the RMC exchange messages in the RMA. These messages follow a text-based protocol, which defines three types of possible formats between RMA layers: Device, Data, and Action.

Before any IoT Object performs the initialization process algorithm, it needs to access a configuration file—in JSON format and defined by the IoT Object’s designer—containing all its information to build the Device message. In this paper, we adopt the *deviceName* as the identifier attribute for identifying an IoT Object in the RMA without knowing its UUID, which is a 128 bits value to be manipulated by clients, users, or designers. In this way, it is easier for clients to find IoT Objects using a direct name instead of UUID. Then, the UUID only addresses the IoT Objects in the IoT network, while *deviceName* is used in RMA. When the RMC receives this type of message, it creates the Device object, updates the VCDB, and gives back an acknowledge message in Device’s message format.

The Data message represents the values sensed by Resources. To build this message, the IoT Object reads the sensor, mounts a Data object, and sends it to RML. When the RMC receives this message, it stores the resources’ measured values into the VCDB to be accessed later by clients’ applications. The Action message represents a request for executing some command by an IoT Object’s actuator. When the RMC receives any client command, it creates an Action object, converts it into an Action message, and sends it to the VCDB. Both Data and Action messages are stored in VCDB to maintain historical data of Resources as a dataset for future data mining and analysis.

Finally, in this refactored RMA, the VCDB uses a NoSQL database (MongoDB) to improve database operations performance. In addition, the messages adopt the JSON format to simplify the communication and the process of exchanging messages. We conducted some tests for evaluating the refactoring performance considering some aspects of how RML deals with connectivity and messages in Section 4.3.

#### 3.3.2. The Communicator Agent Extension

The Communicator Agent [3] is an agent architecture created for the *JaCaMo Framework* [12] that allows agents to exchange messages in an IoT network. However, this agent extension does not deal with the RMA components. Then, we developed a new Communicator agent capable of communicating with RML. It works as the bridge between the IoT Object and the RML. It sends the IoT Object registration request, Data to RML, and receives Actions to be executed in actuators. Every embedded MAS will have only one Communicator agent to uniquely identify the IoT Object in the IoT network and RMA since it is also capable of communicating with other embedded MAS connected in the IoT network, and it needs to be reachable for clients. Finally, it was adapted to deal with the Device’s configuration file to become able of sharing its resources with RML.

The extended Communicator Agent can perform two new internal actions: *connectToRml* and *sendToRML*. Internal actions are pre-defined functions with a specific purpose that agents can perform anytime during their life cycle. These new actions do not affect the physical environment, and they are intended to communicate with RML. Each internal action is detailed as follows:

- *connectToRml*: It is responsible for establishing a connection with RML. It has three parameters, the IP address, the RML serial port, and the Device’s configuration file. When this internal action is called, the connection to the RML is established, and the IoT Object becomes part of the architecture. In fact, it implements the IoT Object’s initialization process described in Algorithm 1.
- *sendToRML*: It is responsible for sending the Resources’ Data to the RML. All this information is stored in the agent’s belief base, where agents keep all their observations, information received by communication, and perceptions. Considering this, Physical agents gather information from Resources and send them to the Communicator agent, which stores the data as beliefs until this internal action is called. If a second Data from the same Resource arrives from Physical agents before the first one has been sent to RML, the internal action will consider the most recently received. For example, if the Communicator agent already has a resource’s Data named *temperatureSensor(cold)* in its belief base and receives a new *temperatureSensor(hot)*, the latter will be selected. It implements the sending behavior described in Algorithm 2 (lines 5 and 6).

When a Communicator Agent connects in the RML, it initializes a listener who will receive all Actions sent by RML and convert them into intentions. In the Belief-Desire-Intention (BDI) model, an intention is a desire that an agent has compromised itself in achieving. All desires are expressed as plans, composed of a serialized steps known as actions—including sending messages to other agents or acting upon the physical environment for example—allowing agents to achieve their goals [32].

Finally, during the Communicator’s reasoning cycle execution, Data are sent to the RML using the *sendToRML* internal action, and Actions are received. They could be forwarded to be executed or not, depending on the agent’s deliberation. This process implements the behavior of the IoT Object’s cycle defined in Algorithm 2.

#### 3.3.3. The IoT Artifact Extension

The Physical Artifact [4] is used by agents to interface with the hardware’s sensors and actuators without affecting agents’ reasoning performance. However, Physical Artifacts do not have support for RMA. Therefore, we extended the architecture of Physical Artifacts for connecting and communicating directly to the RML, without modifying their ability to interface hardware. In addition, agents still access these artifacts for acquiring information and controlling actuators. Every embedded MAS can have more than one IoT Artifact to handle several microcontrollers, but only one will identify the IoT Object in the RML because it needs be reachable by clients.

The IoT Artifact has a new method and two new external actions that can be managed by agents as follows:

- *enableIoT*: This method allows the IoT Artifact to become an IoT Object in the RMA and communicate directly with RML. Thus, it implements the IoT Object’s Initialization Algorithm 1 and Cycle Algorithm 2. For this, it uses the Device’s configuration file—to share its resources with RML—and network address information to initializes the IoT Object in the RML and start its Cycle.
- *percepts*: The external action responsible for reading hardware sensor’s data. It uses a serial interface to gather sensor’s data and convert them into Observable properties—artifacts’ variables that can be perceived by agents. All agents who access this artifacts will have the data converted as beliefs. Finally, it implements the Data buffer building process of IoT Object’s Cycle Algorithm 2 (line 5).
- *act*: It is the external action responsible for sending a command to an actuator using a serial interface. All agents who want to control the actuator will inform the artifact of the Resource’s Command name. Finally, it implements the *onAction* process of IoT Object’s Cycle in Algorithm 2 (line 4).

In the A&A approach, the Communicator Agent is responsible for communicating with RML and Artifacts for interfacing hardware. Nevertheless, in the IoT Artifact approach, the Communicator Agent is no longer used. Instead, the IoT Artifact assumes the control of the information flowing between the RML and the hardware. Even so, agents can still read Data accessing Physical Artifacts’ *Observable properties* and perform actions using the *act* operation.

#### 3.3.4. Technologies Employed

The RMA employs different technologies for integrating all three of its layers. They allow embedded systems to manage and control hardware, IoT Object communication with RML, and client requisitions. Our architecture demands an IoT infrastructure that should be provided by any IoT middleware that deals with device connection and reconnection, message exchanges from clients instances (in our case, IoT Objects and Clients) to the cloud, security issues, and scalability. The IoT middleware plays a major role since it is responsible for the main functionalities that allow virtualization and data consumption by clients. We adopted the ContextNet [14] middleware as the IoT infrastructure for RMA.

ContextNet is a middleware that provides a context service in the publish/subscribe model for large-scale collaborative applications between entities such as smartphones, vehicles, autonomous mobile robots, etc. Its communication and context distribution capabilities are implemented in the Scalable Data Distribution Layer (SDDL). ContextNet also deals with the main data communication issues, such as fault tolerance, network load balancing, support for disconnecting nodes, and security. The data transfer occurs using two protocols: MR-UDP for exchanging messages between the gateway and clients [33] and OMG DDS for data distribution within the network core [34].

The embedded system could also employ any technology available since it complies with the protocol and adopts a client instance of the IoT middleware chosen. In our case, we chose the *JaCaMo* framework as the cognitive embedded system because it has two dimensions used by IoT Objects—Agents and Artifacts—and provides an organizational model [35] that could be used in future approaches. We adopt the Javino [36] as the serial interface for accessing sensors and actuators since it was developed specifically for embedded systems for Java and *JaCaMo*.

Both RML and Client’s application adopts ContextNet as part of their implementation. The former uses a server instance to receive data messages from IoT Objects and actions from clients. The latter just uses a client instance for sending actions. All data consumed by clients are retrieved directly from VCDB, which uses a NoSQL database, the MongoDB.

## 4. Experimental Evaluation

The three proposed engineering approaches aim to provide more autonomy by adopting embedded MAS in the edge of an IoT system using the RMA. Since the RMA constructions and all approaches are intended to run on real devices in physical infrastructure, we present a study case in a home garden scenario where all characteristics proposed in this paper are implemented in hardware, presented, and discussed. After that, we perform some latency tests to evaluate if there is any difference by adopting one or another approach considering our scenario.

### 4.1. A Home Garden Scenario

The essential resources for a plant to survive are water and light, but for any plant to reach a high level of productivity, these resources must be adequately balanced not to lack or exceed. Other aspects must also be observed when cultivating plants, such as the soil pH level and soil moisture. All this information can be used to understand whether the plant will survive and bear fruit or wilt and die. Thus, in a scenario where an inexperienced user is trying to start a vegetable garden in his house, a device that gathers essential information about the garden environment could help this user once it is possible to obtain all the available information from a distance and also interact with this garden.

An inexperienced user could also forget to water the plants or could be too busy to do that. In this case, a cognitive system embedded in a device could bring some advantages since it can obtain information, process it, and make the necessary changes autonomously, benefiting the home garden. It can makes the plants’ care easier by making human intervention unnecessary to perform simple tasks. In addition, any user could also be far from his garden in a critical moment, for example, if the day is too hot and it is necessary to turn on the irrigator manually. Then, functionalities in this home garden for controlling actuators and verifying information remotely would be interesting and necessary. For this to happen, the device must be connected to some network, making it possible to share data, make it available to the user, and allowing the user to send commands to be executed by the device. Thus, this user will adopt the RMA for managing its home garden.

**Figure 4:**
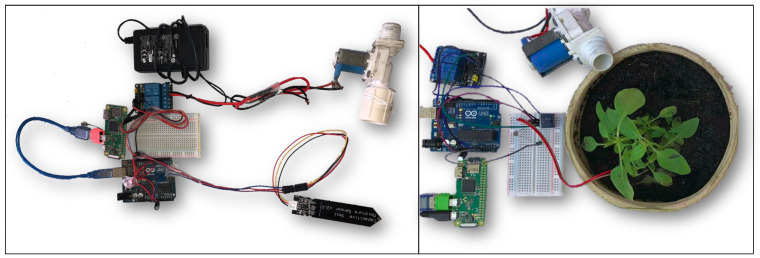
The IoT Object in the garden scenario is composed of an actuator for irrigation and sensors to measure soil’s pH level, soil moisture, luminous incidence, and temperature.

**Figure 5:**
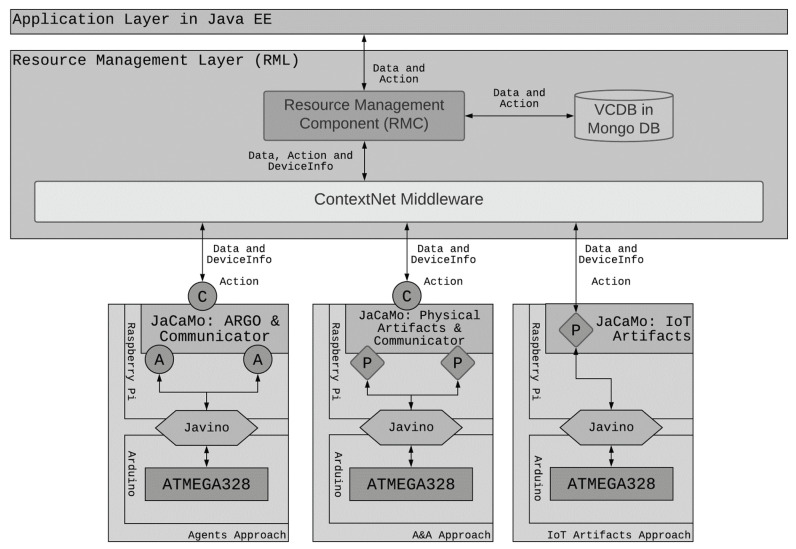
The technological view of components considering the IoT Objects employed in tests and the Engineering approaches in extended RMA.

Therefore, we assembled an IoT Object composed of an actuator for irrigation and four sensors to measure the soil’s pH level, soil moisture, luminous incidence, and temperature (Figure 4). The sensors and actuators are connected to an ATMEGA 328 microcontroller, which in turn is connected to a Raspberry Pi Zero. This board stores the Embedded MAS that interfaces hardware and communicate with the RML. All the data collected by the MAS are used for the decision making that aims to guarantee the home garden’s survival. In all engineering approaches, this decision process is performed by agents in the IoT Object. In the Agents and A&A approaches, even if the home garden owner sends actions, agents can deliberate before redirecting these actions to the microcontroller. For example, if the soil is already wet, it is not necessary to irrigate again. However, in the IoT Artifact Approach, the user has more control, and agents do not interfere in Actions, which will all be executed by the IoT Artifact. The IoT Object’s technological architecture is seen in Figure 5.

We adopt this home garden scenario as Proof-of-Concept of the engineering approaches presented. For this, one embedded MAS was implemented for each approach using the IoT Object hardware configuration (presented above). Moreover, the RML was executed in a regular machine, with an i7 processor and 8 GB of RAM. The IoT Object was executed in a Raspberry Pi Zero, with a Broadcom BCM2835 of 1 GHZ and 512 MB of RAM. For the scenario, all the clients’ Actions were performed using a Graphical User Interface (GUI) in JavaFX to create Actions in VCDB.

### 4.2. The Embedded Processing Cost Tests

The scenario ran as proof-of-concept worked as expected, achieving the purpose of managing a home garden autonomously by the IoT Objects and dealing with some user’s actions when necessary. However, it is important to observe that the Embedded MAS running on top of IoT Objects could influence the system performance. In fact, knowing the impact that an intelligent system on the edge of an IoT system causes could help designers and programmers choose the best approach to tackle certain domains. One way is to measure the cognitive system processing cost during the execution of the scenario.

Considering this, during the proof-of-concept execution, we considered the following business rules: the temperature cannot be below 10 ${}^{\circ }$C or above 35 ${}^{\circ }$C to ensure the plantation will stay alive in the home garden scenario, and the Ph level must be between 5 and 5.8. In addition, the moisture must be at an acceptable level to not drown or dry the plant. The Embedded MAS uses these business rules to decide when to turn on the sprinkler or send an alarm to the client. The MAS will turn on the sprinkler when the agent perceives the soil as dry and will turn it off when the soil is wet or raining. Finally, the MAS notifies the clients about the sprinkler operation status and the moisture, Ph, and temperature status (whether it is out of range or not).

The Embedded MAS Processing Cost (EMPC)—representing the time agents and artifacts from the Embedded MAS take to interface hardware, reason, and communicate—is calculated differently for each approach, considering Physical and Communicator agents, Physical Artifacts, and IoT Artifacts. The EMPC includes the time from the hardware collected data until the message is sent to the RML. Each approach implements the business rules described previously and has its EMPC measured based on 200 messages sent to RML. The Table 2 shows the results obtained based on the following configuration:

1. Agent Approach: It employs one Physical Agent using ARGO, a JaCaMo extension [37] for interfacing hardware and collecting information from the home garden; a mediator agent, which receives the information collected from the Physical Agent and processes them; and one Communicator Agent, which receives the information from the mediator and sends to the RML. The EMPC is composed of the Physical, Mediator, and Communicator Agents’ processing cost. The time capturing begins when the Physical Agents gather hardware perceptions and stops when the Communicator Agent sends information to the RML.
2. A&A Approach: It uses a Physical Artifact to collect data from the physical environment; one Mediator Agent, which accesses the Physical Artifact and processes information collected; and one Communicator agent, which receives the information from the mediator and sends it to the RML. The EMPC is composed of the Data arrival at the Physical Artifact, the Mediator Agent capturing and reasoning cost, and the Communicator cost of sending the information to the RML.
3. IoT Artifact Approach: It employs a Physical Artifact to collect and send the data to RML and a MAS to process the data and make decisions locally. The MAS analyzes the data and interfaces only with the Physical Artifact, not interfering in collecting or sending data to the RML. For this approach, the EMPC is calculated considering the Data arrival at the Physical Artifact and when Data leave this artifact.

**Figure 6:**
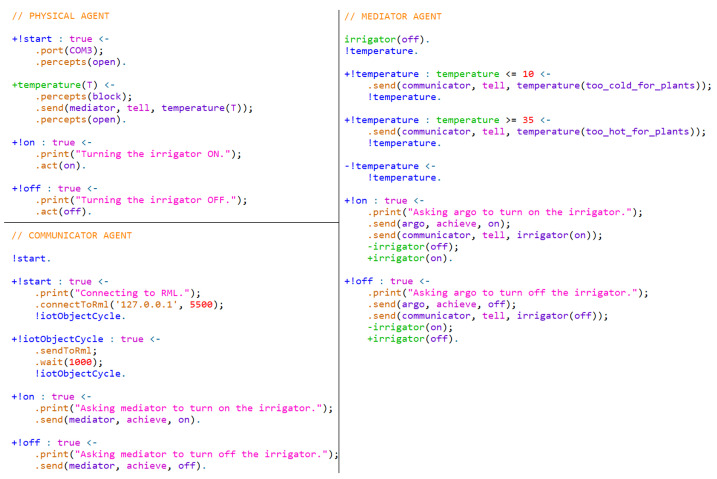
The Agent Approach code.

**Figure 7:**
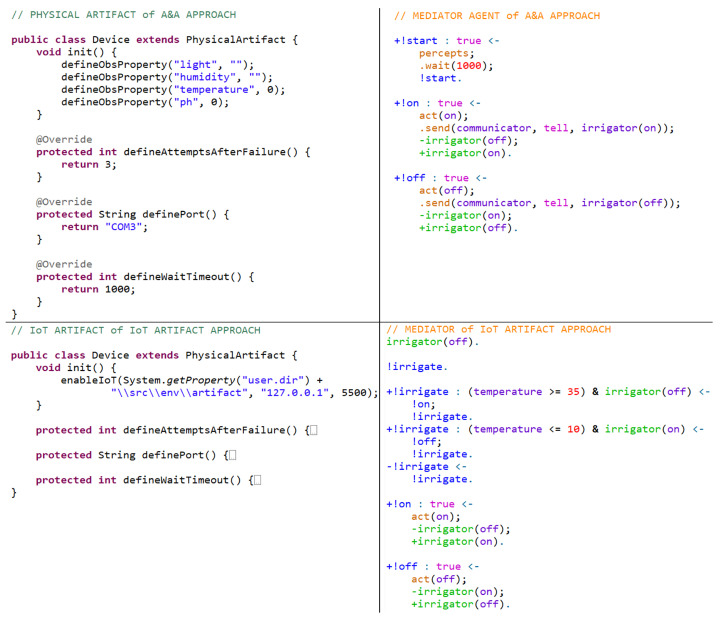
The A&A and IoT Artifact Approach codes.

The codification of both agents and artifacts uses all traditional commands from JaCaMo and those presented in this research. In the Agent Approach, the Physical agent uses the customized internal actions to open the perceptions capture and select which serial port to access. In addition, it can act directly in hardware by using the internal action *act*. The mediator agent is a typical agent who deals with all the information received from the Physical and Communicator agents. The Communicator agent uses the *sendToRml* new internal action to send information from its belief base to the RML. It also connects to the RML by using the *connect to RML* new internal action. Considering the hardware and RML interface, everything else is transparent for the IoT designer and MAS programmer. Figure 6 and Figure 7 show an adapted and reduced version of the implementation.

In the A&A Approach, there is no Physical agent. Only the Mediator and Communicator agents exist, but the former one is slightly different. The difference is that the Mediator agent is configured to access the Physical Artifact to update the beliefs by accessing the external action *percepts* and activate actuators by accessing the external action *act*. The Communicator agent remains the same. In the IoT Artifact Approach, the Mediator agent is responsible for accessing the IoT Artifact and acting upon it if necessary. There is no need to employ communicator agents since the data transmission is in charge of IoT Artifacts. In addition, the IoT Artifact also performs hardware interfacing. Using Physical or IoT Artifacts is the same as using typical artifacts in JaCaMo. All hardware interfacing and data transmission are transparent from the point of view of agents.

Comparing the Agent and the A&A approach, physical agents are the most costly because they need to interface hardware, process the captured perceptions as beliefs, and deliberate which plan activate while performing its reasoning cycle. An artifact does not have to deliberate which plan activates based on perceptions coming from sensors. It is accessed by any agent who needs to access data or perform actions using actuators. In addition, in the Agent approach, the Communicator agent receives data from Physical agents and processes them as beliefs, whereas in the A&A approach, it accesses the artifact to gather the available information also as beliefs. In both cases, the cost is practically the same. The EMPC from approaches that use agents (Agent and A&A approach) doubles compared to the IoT Artifact approach because, in the latter one, artifacts send data directly to the RML without any processing or interference from the MAS.

Analyzing the approaches and their applicability, the Agent and the A&A approaches are suitable when the system needs more autonomy in the edge, and improved data must be sent to RML instead of raw data. Non-cognitive systems usually send just raw data, which is the exact value coming from sensors. Agents can improve this information by reasoning upon it and getting into an elaborated conclusion. For example, a temperature sensor provides a value, but it does not inform if it is hot or cold. The main difference between these approaches is that, in the Agent approach, dedicated agents deal with hardware interfacing and reasoning (centralized behavior). In the A&A approach, all agents from the Embedded MAS can access the available artifacts if they are not busy (decentralized behavior). The IoT Artifact approach is applicable when there is a need to send data directly to the RML, but it is still important to maintain MAS interference in the edge locally (independent behavior).

### 4.3. The RMA Performance Tests

The RMA performance tests calculate the elapsed time between the IoT Object and RML’s communication. The objective is to analyze the communication and processing latency of the extended RMA by implementing the new algorithms and a NoSQL database. The results of this test will be discussed and compared with the classic RMA (presented in Section 3) performance tests.

We created 50 simulated IoT Objects and 1 RML server. We obtained the timestamps for sending messages to the RML, the RML reception, and VCDB data registering for each IoT Object. These messages can come from the initialization process or the Data buffer of the IoT Object’s cycle. The test produced two different results: messages generated with a one-second time interval and a five-second time interval. For each result, 3500 messages were registered.

**Figure 8:**
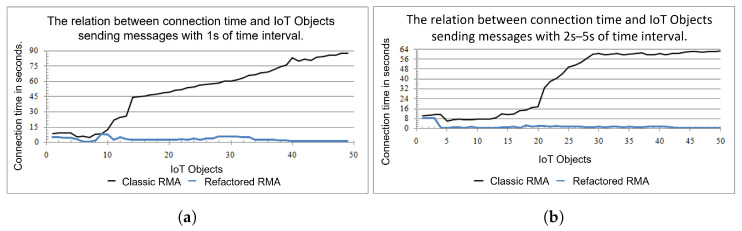
Connection time comparison between the RMA and the refactored RMA: (**a**) considering 1 s interval of messages; (**b**) considering considering an up to 5 s interval of messages.

**Figure 9:**
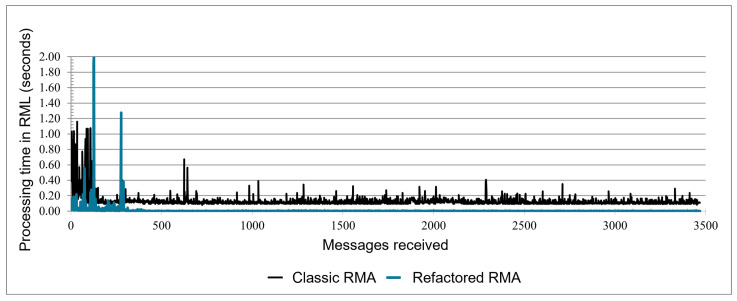
The comparison between RML’s message processing time.

We built the same correlations performed for the classic RMA from the test result to analyze and compare them. The test result is shown in three charts: one relating the number of IoT Objects with the time of sending messages to RML adopting a one-second time interval (chart a from Figure 8), another one doing the same relation but with a five-second time interval (chart b from Figure 8), and the last one relating the RML processing time in seconds with the total amount of messages arrived at it (Figure 9).

Figure 8 shows two line charts (one-second and five-second time intervals) relating the connection time in seconds—ordinate axis—and the number of IoT Objects sending messages to RML—abscissa axis. In both, two series represent the time in seconds as a function of the IoT Objects’ number, the black series being the results of Classic RMA and the blue series the Refactored RMA. Figure 9 shows the chart relating the processing time in second in the RML with 3500 messages received. This chart also contains two series: the black for the Classic RMA and the blue for Refactored RMA.

Considering the IoT Objects connecting themselves in RML and then sending messages with a time interval of 1 s, as the IoT Objects request to connect, they obtain a fast response since few IoT Objects are trying to connect and send messages that the RML must deal with. Since it is the first time that IoT Objects are requesting connections, there will not be competition once the number of messages and IoT Objects treated by RML are low. Once every IoT Object is connected, it starts sending messages, and the RML has to deal with both connection requisitions and message processing. As more IoT Objects connect in RML, the time response increases linearly, as seen from 10 to 50 in Figure 8a. There is some instability in the IoT Objects’ connection in the Refactored RMA as they request and start to send messages. However, the time response does not grow over as more IoT Objects become part of the system, and it tends to stay so proving that this new refactored version of RMA overcomes one of the main concerns of adopting the proposed architecture, the RMA scalability.

When the time interval is increased up to 5 s, there is no difference from the former analysis. In the Classic RMA, as the IoT Objects connect and send messages, the response time increases significantly—from 20 to 30 IoT Objects—and then stabilizes in about 60 s. In the Refactored RMA, the response time from the RML started at 8 s and then decreased to below 1 second, and then it stabilizes to about 1 s, as seen in Chart b from Figure 8. The connection response time also tends to stay the same. However, stressing the RMA with up to thousands of IoT Objects could give a better perspective of the range of a system adopting the architecture. For instance, we assert that the RMA is suitable for domains employing 50 to 100 IoT Objects since most of them do not need to send messages all the time. First, because there is an Embedded MAS to decide whether to send some information to RML, the information can take some time to change, for example, the temperature or if it is raining or not.

Another characteristic observed was the message processing time in RML. Every message that arrives from IoT Objects in RML is processed as a registering request or Data message. Connections requests take more time to be processed than messages since all resources and IoT Object basic information must be inserted in VCDB if it is the first time the IoT Object registers in the RML. The Classic RMA takes about 1 s to process the first messages, which includes the connection requests. The Refactored RMA takes a half-second for the same processing, but peaks over 2 seconds occur. After all IoT Objects have connected in RMA, the processing time stabilizes to about 0.2 s for the Classic RMA and below 0.1 in the Refactored RMA. It is important to remark that a stack of messages is to be processed as IoT Objects connect and start sending messages. Moreover, there is the network latency between IoT Objects and RML. However, the processing time associated only with RML is satisfactory, and it tends to stay so if the messages continue to increase, as seen in Figure 9.

## 5. Final Considerations Include (in a Point-Wise Manner) 3–5 Main Findings of This Research

This paper presented three engineering approaches using embedded MAS in IoT Objects supported by an extended IoT architecture for devices virtualization. This embedded cognitive system can reason to provide autonomy and pro-activity to the IoT Object at the edge of the system. Adopting a whole embedded MAS into an IoT Object is not a simple task since it is necessary to provide interfaces for hardware control, IoT communication, and communication with other IoT Objects. Actually, traditional approaches use one agent to control all devices’ functionalities or centralized approaches, which imply high dependency. This paper presented approaches for programming embedded MAS using JaCaMo framework extensions capable of creating communicator agents able of sending data to an IoT server and exchanging messages with other IoT Objects and agents to interface hardware. As JaCaMo allows programming the agent’s endogenous environment dimension by using artifacts, two extensions of artifacts were presented for controlling hardware and sending perceptions directly to the RML of the proposed architecture.

The three engineering approaches aim to facilitate and diversify the designer’s choice in how to assemble and employ the IoT Objects in physical environments considering some characteristics such as heterogeneity, hardware availability, latency, data availability, and user’s needs. In the Agent approach, the agents of the embedded MAS are responsible for all IoT Object’s behaviors, from capturing sensors’ data to sending them to the RML. It provides a high level of autonomy and pro-activity to the device and allows it to interfere pervasively in the physical environment without depending on other layers. However, sometimes agents that interface hardware could be overloaded if the number of sensors and actuators grows. In the A&A approach, the physical artifacts presented in this paper collect information from sensors, and the MAS is responsible for reasoning and delivers it to the RML. It provides democratic access to sensors and actuators since any agent can access an available artifact.

However, the communication is still specialized, where the proposed communicator agent is responsible for interfacing with the RML in the IoT network. It still provides autonomy and pro-activity to IoT Objects removing possible bottlenecks in accessing hardware, but only one agent responsible for all communications with the RML and other IoT Objects could be overloaded, as well. Finally, the IoT Artifact approach uses physical artifacts to collect and send information to the RML. The embedded MAS is responsible only for acting at the edge of the system for guaranteeing autonomy and pro-activity. The information is sent directly to the RML in a raw format, without any reasoning or interference of agents. It loses processing and reasoning but transfers to the upcoming layers the interpretation of the data collected.

The advantage of using the Agent approach is to specialize agents to overcome processing bottlenecks compared to single agent-based implementations. It is recommended as an embedded system when few agents need to receive sensor data and there is no competition for accessing the same sensors. By adopting the A&A approach, any agent can access the physical artifact and any competition the artifact itself can deal with. In both the Agent and the A&A approaches, the sensors data need to get to the agents before getting to the RML, but agents can reason upon the raw data to produce a better and more comprehensive perception. They are recommended when understanding the environment is a priority. Last but not least, the IoT Artifact approaches sends sensors’ data directly to RML, prioritizing the data availability instead of understanding.

We presented a case study using a home garden scenario to measure and analyze the processing cost of the proposed engineering approach, and we also considered how long the message takes to reach the RML and its processing cost. We considered all the Data transitions and processing in all three approaches—from hardware to specialized agents and artifacts and IoT Artifacts—for understanding the behaviors of each one of them. In addition, a comparison is shown between this version and the previous one. From the tests, it was possible to state that the engineering approaches are suitable for implementing embedded MAS at the edge of an IoT system. Moreover, the presented refactored RMA proved to be more efficient and scalable considering the response, processing time, and projections.

For future works, it is possible to adapt the RMA for creating a mechanism for transferring plans between the embedded MAS of IoT Objects. Since BDI agents are capable of learning new plans at runtime—especially when using JaCaMo—a new type of message could be created to address plans. In this case, any agent with a particular set of sensors could ask for some plan implementation to any IoT Object with the same set of sensors. It is an interesting issue when considering that plans could evolve during the lifetime of IoT Objects. Therefore, another issue that the RMA could address is Planning. A mechanism for creating new plans or refining and improving existing ones based on past experiences and user preferences could extend the applicability of embedded MAS and IoT in some domains. For example, in Industrial applications, it is essential that the manufacturing does not stop for some maintenance and setups, or it could be costly to perform a software update in hardware, e.g., deep underwater hardware. Another possibility is also to use Machine Learning in the RML to generate plans for the embedded MAS. All these generated and improved plans could also be shared between IoT Objects. 

## Figures and Tables

**Table 1:** Comparison between the related works.

Work	Platform	Domain	Agent Composition
[16]	Jade	Crop Irrigation	Agent per Device
[18]	Hardware and Android	Smart Home	Agent per Device
[19]	Jade	Robotics	Agent per Device
[20]	CVL—SelfStar MAS	Software Product Line	Agent per Device
[22]	PANGEA	Catlle	Agent per Device
[23]	ATMega and ESP8266	Co-working	Agent per Device
[26]	Jade	Vehicles	Agent per Device
[27]	Jade	Wireless Sensors Network	Agent per Device
[28]	Jade	Generic	Agent per Device
[29]	Jade	Generic	Agent per Device
This	JaCaMo	Generic	Embedded MAS

**Table 2:** Performance tests’ results of the engineering approaches after 200 messages sent.

	Physical Agent		IoT Artifact		Comm. Agent		EMPC	BEMPC
	avg	sd	avg	sd	avg	sd		
**Agent**	1.6452	0.5207	-	-	3.2457	1.3523	4.8910	3.9998
**A&A**	-	-	0.4209	0.2134	3.6688	1.0355	4.0897	1.7628
**IoT Art.**	-	-	2.0438	0.9211	-	-	2.0438	2.0438

## Data Availability

The RML developed in Java is available at https://github.com/TuringResearch/rma-Java (accessed on 26 October 2021). The JaCaMo extension for programming Argo and Communicator agents, Physical Artifacts, and IoT Artifacts are available at https://github.com/TuringResearch/jacamo-4-rma (accessed on 26 October 2021). All details for installing the employed technologies are described in the *readme* of each repository. The performance test results files are also available for download in the *readme* file of the test branch of each project.
